# Perceptions of veterinarians and producers concerning Johne’s disease prevalence and control in US beef cow-calf operations

**DOI:** 10.1186/1746-6148-10-27

**Published:** 2014-01-23

**Authors:** Bikash Bhattarai, Geoffrey T Fosgate, Jason B Osterstock, Seong C Park, Allen J Roussel

**Affiliations:** 1 Department of Veterinary Integrative Biosciences, Texas A&M University, College Station, TX 77843, USA; 2 Department of Production Animal Studies, University of Pretoria, Onderstepoort, South Africa; 3 Zoetis, Kalamazoo, MI 49007, USA; 4 Texas A&M AgriLife Research and Extension Centre, Vernon, TX 76385, USA; 5 Department of Large Animal Clinical Sciences, Texas A&M University, College Station, TX 77843, USA; 6 Current address: Institute of Biomedical Studies, Baylor University, One Bear Place, Box 97261, Waco, TX 76798, USA

**Keywords:** Johne’s disease, Beef cattle, *Mycobacterium avium* subspecies *paratuberculosis*

## Abstract

**Background:**

Efforts to educate producers and veterinarians in the United States regarding the management, prevention and control of *Mycobacterium avium* subspecies *paratuberculosis* (MAP) infection have increased over recent years. While nationwide awareness about MAP infection is improving, current level of awareness among beef producers and veterinarians is largely unknown. This study compares the perceptions of beef producers and veterinarians on the burden of MAP infection in cow-calf herds and on measures to control new infections. Questionnaires were mailed to 989 US beef producers through state Designated Johne’s Coordinators and to 1080 bovine veterinarians belonging to a US nationwide professional association.

**Results:**

Twenty-two percent (34/155) of producers reported having infected animals in their herds. The mean (minimum, median, maximum) prevalence reported by producers was 0.8% (0, 0, 10). Twenty-seven percent (27/100) of producers had at least one clinical animal during the previous year. Compared to the small herds (<50 head), the mean test-positive percentages and estimated prevalences were higher in medium (50–149) and highest in large (≥150) herds. Seedstock herds had a lower prevalence and these producers were more likely to enroll in Johne’s disease (JD) control programs and test their herds. Veterinarians reported a mean overall animal level prevalence in their client herds of 5% (0, 2, 60). Similarly, 26% (0, 10, 100) of client herds had at least one infected animal. Mean percentage of infected cows within infected herds was 9% (0.01, 5, 80). Producers generally performed activities to control MAP transmission more frequently than perceived by veterinarians. Compared to veterinarians’ opinions, producers were less likely to cull cows with signs consistent with JD (P < 0.01), but more likely to test purchased additions (P < 0.01). Testing recommendations by veterinarians (n = 277) for beef cow-calf herds were bacterial culture of feces (3%), PCR (14%), ELISA (35%) and a combination of these tests (47%). Seventy-nine percent of veterinarians recommended a 12-month interval between testing.

**Conclusions:**

Seedstock producers who had had JD risk assessments performed on their farms were more supportive of JD control programs and had a correspondingly lower prevalence. It is important to increase educational activities to provide relevant information to veterinarians and producers for better management and control of JD. Educational programs should target larger herds to maximize the impact.

## Background

*Mycobacterium avium* subsp. *paratuberculosis* (MAP) is the etiologic agent associated with bovine paratuberculosis, which is commonly known as Johne’s disease (JD). In cattle, JD is characterized by a chronic granulomatous ileocolitis, a long pre-clinical phase terminating in diarrhea, debilitation [1,2], cachexia and death [3]. Reported prevalence estimates of MAP infected beef cattle in the United States vary widely, ranging from 0.4% to 9% of animals and 8% to 63% of herds based on detection of MAP-specific serum antibodies [4-8].

The Beef’ ’97 and Beef 2007–08 studies by the National Animal Health Monitoring System (NAHMS) estimated that 92% and 69%, respectively, of beef producers were unaware of JD or only recognized the name without having direct knowledge about the disease [4]. The United States Voluntary Bovine JD Control Program (VBJDCP) was implemented since 2003 to provide minimum national standards for the control of JD and to educate veterinarians and producers regarding management, prevention and control of JD [9]. A survey in Texas during 2006 suggested that only 20% of beef producers were familiar with the VBJDCP and 16% considered participation [10]. Sixty-four percent of veterinarians in Texas had educated beef producers on management strategies for the control or elimination of JD. However, only 36% of these veterinarians had received specific training regarding JD epidemiology and 29% were JD-certified [10].

The VBJDCP has been the official control program developed in cooperation with industry stakeholders and experts and subsequently approved by the United States Department of Agriculture. The US government provided financial support for the control program and to assist producers with risk assessments, management changes, and diagnostic testing [11]. The VBJDCP consists of three elements: education, management, and herd classification [12]. An important component of the education component is veterinarian certification through an Internet-based training. The management component is implemented by veterinarians certified to perform JD risk assessments and develop management plans. A JD risk assessment is a structured investigation of management practices based on an on-farm evaluation of risk factors and owner responses related to management practices and biosecurity [9,13].

The objective of this study was to compare the perceptions of beef producers that had risk assessments and herd management plans developed for JD and bovine veterinarians on the burden of MAP infection in cow-calf herds and measures to control new infections. A secondary objective was to compare perceptions among different types of cow-calf producers and veterinarians.

## Methods

The study protocol was approved by the Institutional Review Board at Texas A&M University (protocol number 2010–06666).

### Questionnaire

A mailed questionnaire was administered to beef producers in 9 US states (FL, GA, IA, MO, ND, SC, SD, WI, WV) that had risk assessments and herd management plans developed for JD. At the time of the survey, producers were either actively participating or had participated in the past in a JD control program. The Designated Johne’s Coordinators of each selected state mailed questionnaires to all beef producers that had had JD risk assessments performed and herd management plans developed for JD.

Another questionnaire was mailed to veterinarians from the same 9 states with active membership in a nationwide professional organization and who listed “bovine” as one of their practice types. The majority of questions for the veterinarian and producer questionnaires were comparable. Veterinarians were contacted with an introductory letter 12 days prior to the mailing of questionnaires. A business reply envelope and a $2 bill were included in each questionnaire packet as an incentive to improve response proportions [14]. Reminder post-cards were mailed 8 days after the questionnaire. Additional details on questionnaire development and administration have been described elsewhere [15].

### Analysis

Responses from completed questionnaires were recorded on a secure server using SelectSurvey (ClassApps.com, 2006, SelectSurvey.NET 1.5.1). Data were analyzed using Stata® (StataCorp. 2011. Stata® Statistical Software: Release 11.2. College Station, TX: StataCorp LP) and OpenEpi [16]. Continuous outcomes were described using the mean, minimum, median, and maximum values. Variables were evaluated for a normal distribution using the Shapiro-Wilk test. Wilcoxon rank-sum tests were used to compare variables that were not normally distributed. Two-sided statistical tests were performed and results were interpreted at the 5% level of significance.

To account for variation in response among questions, response proportions were estimated using the number of total respondents to the specific question as the denominator. Responses regarding whether the herd was ever tested for JD or currently enrolled in any JD control program were compared between respondents with and without specific herd types. Burden of MAP infection in producer herds and veterinarian client herds were summarized and compared among types of herds. Likert scale responses concerning the frequency of selected disease control activities performed by producers and perceived by veterinarians were dichotomized. The “always” and “mostly” categories were collapsed into a single “yes” category and the categories “seldom” and “never” were collapsed into “no”. The category “sometimes” was handled as missing data. Frequencies of these activities performed with the intent of controlling JD were evaluated using odds ratios with corresponding 95% confidence intervals and mid-point exact P values. Three herd size categories were created with each group having an approximately equal number of observations: small (< 50 head), medium (50–149 head) and large (≥ 150 head) herds.

## Results

### Description of producers

Questionnaires were mailed to 989 cow-calf producers. Twenty-four questionnaires were undeliverable due to incorrect addresses. The response proportion was 17% with 160 questionnaires returned. The mean (minimum, median, maximum) herd size reported by producers was 155 head (3, 70, 2500). Thirty-five percent (54/155) of herds had less than 49 adult cows, 35% (55/155) of herds had 50 to 149 cows and 30% (46/155) of herds had 150 or more cows. Mean number of years in the cow-calf business was 32 (6, 32, 83). Angus was the predominant breed reported by 52% (81/156) of respondents. A total of 58% of producers had commercial cow-calf herds and 56% were partially or fully comprised of purebred seedstock (Table 1).

**Table 1:** Description of the herds of producers (n = 160) and client herds of veterinarians (n = 325) who responded to the Johne’s disease questionnaire

**Variables**	**Producers**	**Veterinarians**
**Number (%) of herd types**		
Commercial cow calf^a^ - registered^b^	37 (23.1)	224 (70.2)
Commercial cow-calf not registered	64 (40.0)	275 (86.2)
Seedstock^c^ registered	86 (53.8)	189 (59.3)
Seedstock not registered	10 (6.3)	107 (33.5)
Feedlot^d^	0	184 (57.8)
Backgrounder^e^, stocker and dairy herds	0	64 (20.1)
**Herd attributes**		
Mean herd size (min, median, max)	155 (3,70,2500)	105 (0,50,3000)
Percentage of infected herds	21.9	26.3
Mean prevalence (min, median, max)	0.8 (0,0,10)	4.8 (0,2,60)

^a^A cow-calf herd is a beef cattle herd maintained to produce beef calves, which are usually sold after weaning.

^b^Registered herd is registered with a cattle breed association.

^c^Seedstock herds produce cattle of a known pedigree that are typically purebred.

^d^Feedlot is a feeding operation that raises beef cattle until they are finished and ready for slaughter.

^e^Backgrounders and stockers raise calves from weaning until being sent to a feedlot.

Ninety-five percent (149/157) of producers had tested their herds for JD at least once and 74% (117/158) of producers were currently enrolled in either the VBJDCP (89%, 100/113) or had a JD control program designed by their veterinarian. The mean number of years since the inception of a control program was 7 (1, 6, 30). Thirty-eight percent (56/149) of respondents maintained a closed herd. Seedstock producers were 4 times more likely (P < 0.001) to be currently enrolled in a JD control program compared to non-seedstock producers, despite the fact that both groups had risk assessments as defined in the survey sample frame. Enrollment was 10 times less likely (P < 0.001) in producers with commercial cow-calf herds, and 3 times less likely (P = 0.031) for producers with JD clinical cows (Table 2).

**Table 2:** Involvement of US beef cow-calf producers in Johne’s disease testing, enrollment in a control program and maintenance of a closed herd during 2010-2011

**Variable**				**Odds ratio**	**95% CI**	**P**^**a**^
**Tested herd for MAP**		Yes	No			
Commercial cow-calf	Yes	85	7	0.19	0.02 to 1.58	0.099
	No	64	1			
Seedstock	Yes	86	3	2.28	0.52 to 9.87	0.292
	No	63	5			
Closed herds	Yes	52	4	0.59	0.14 to 2.49	0.499
	No	87	4			
With clinical cow	Yes	23	4	0.25	0.05 to 1.20	0.101
	No	69	3			
**Enrolled in a control program**		Yes	No			
Commercial cow-calf	Yes	56	37	0.10	0.03 to 0.30	< 0.001
	No	61	4			
Seedstock	Yes	76	13	3.99	1.87 to 8.53	< 0.001
	No	41	28			
Closed herds	Yes	42	14	1.12	0.52 to 2.39	0.780
	No	67	25			
With clinical cow	Yes	14	13	0.35	0.14 to 0.89	0.031
	No	55	18			
**Maintained a closed herd**		Yes	No			
Commercial cow-calf	Yes	37	50	1.68	0.84 to 3.33	0.145
	No	19	43			
Seedstock	Yes	31	54	0.90	0.46 to 1.75	0.749
	No	25	39			
With clinical cow	Yes	8	15	0.98	0.36 to 2.64	0.975
	No	24	44			

^a^Mid-point exact P values.

### Description of veterinarians

Three percent (31/1080) of mailed questionnaires were undeliverable due to incorrect addresses. A total of 382 questionnaires were returned but 57 lacked useful information. The most common reasons for not completing the questionnaire were that respondents were not currently involved in beef practice (n = 24), there were no cattle (dairy or beef) clients in the practice (n = 23) and the respondents were retired (n = 20). Twenty-eight of these veterinarians also returned the $2 incentive along with the questionnaire. Thirty-one percent of mailed questionnaires provided information that could be used for analysis.

Forty-one percent (132/325) of veterinarians reported that they currently are or were JD certified and 38% (121/317) had performed a JD risk assessment. Risk assessments for JD were performed by 62% (78/126) of JD certified veterinarians and by 22% (42/190) of veterinarians that had never been certified. Eighty-eight percent (279/318) of veterinarians currently served cow-calf herds and 7% (22/318) of respondents had had cow-calf clients in the past. Mean number of herds served by respondents as the primary veterinarian was 58 (0, 30, 1000). Mean size of herds currently served by veterinarians was 105 (0, 50, 3000).

A total of 65% (200/306) of veterinarians reported Angus as the predominant breed in client herds and another 19% (59/306) listed Angus as the second most common breed. Unregistered cow-calf operations (86%; 275/319) were the most frequent type of herd served by veterinarians followed by registered commercial cow-calf (70%), registered seedstock (59%), and unregistered seedstock operations (34%). Feedlot operations were clients of 57% (184/319) of veterinarians and 20% (64/319) of veterinarians also had backgrounder, stocker or dairy clients.

### Burden of MAP infection

Twenty-two percent (34/155) of producers reported having infected animals at the time of questionnaire completion. Mean prevalence reported by producers was 0.8% (0, 0, 10). The basis of estimation for the percentage of infected animals by producers was personal experience (n = 51), veterinarian’s opinion (n = 51), and an extrapolation from local and regional data (n = 4). Of the producers who wrote free-text responses, 87% (110/127) also mentioned a formal testing process as the basis for estimation. In tested herds, the mean reported apparent prevalence was 2% (0, 0, 100). A total of 27% (27/100) of producers had at least one animal with clinical signs suggestive of JD during the previous year. The mean frequency of clinical animals calculated using the number of reported clinical animals divided by the herd size was less than 0.01% (0, 0, 0.2).

The odds of testing the herd for MAP and enrolling in a control program were lower for commercial cow-calf herds compared to other producer types. Relative to non-seedstock herds, seedstock herds had a higher percentage of test positive animals (P = 0.005, Table 3), but a lower percentage of clinical cows (P = 0.045). Compared to herds not enrolled in a control program, enrolled herds had a lower percentage of test positive cows (P < 0.001) and a lower percentage of clinical animals (P < 0.001). The mean producer estimated prevalence was higher in medium (50–149 head) and highest in large (≥ 150 head) herds. Relative to small herds (< 50 head), the odds of having a clinical animal were 4 times higher (P = 0.045) in medium and 7 times higher (P < 0.001) in large herds.

**Table 3:** **Burden of *Mycobacterium avium *** **subspecies *paratuberculosis *** **infection in cow-calf operations reported by US beef cow-calf producers**

**Herd type**	**Responses**		**Median (IQR) of non zero responses (%)**	**Range of all responses (%)**	**P**^**a**^
	**Total**	**Non-zeros**			
**Prevalence**					
Seedstock	86	17	2 (1 to 4)	0 to 10	0.381
Non-seedstock	69	17	3 (1.5 to 5)	0 to 10	
Closed herds	55	9	1 (0.5 to 4)	0 to 8	0.184
Open herds	90	22	2.8 (1.5 to 5)	0 to 10	
Enrolled herds	114	23	2 (1 to 4)	0 to 8	0.287
Not-enrolled herds	41	11	4 (1 to 10)	0 to 10	
**Clinical animals**					
Seedstock	53	10	0.010 (0.006 to 0.017)	0 to 0.04	0.045
Non-seedstock	44	16	0.013 (0.005 to 0.030)	0 to 0.2	
Closed herds	32	8	0.007 (0.006 to 0.08)	0 to 0.031	0.883
Open herds	59	15	0.014 (0.005 to 0.033)	0 to 0.2	
Enrolled herds	67	13	0.011 (0.006 to 0.016)	0 to 0.111	0.011
Not-enrolled herds	30	13	0.015 (0.006 to 0.029)	0 to 0.2	
**Test positive animals**					
Seedstock	80	13	1.5 (1 to 3)	0 to 100	0.005
Non-seedstock	55	21	2 (1 to 5)	0 to 100	
Closed herds	49	10	1.1 (1 to 5.5)	0 to 100	0.300
Open herds	78	22	2 (0.8 to 5)	0 to 100	
Enrolled herds	105	18	1.75 (1 to 4)	0 to 100	< 0.001
Not-enrolled herds	30	16	1.8 (0.9 to 5.25)	0 to 100	

^a^Wilcoxon rank-sum test based on all responses.

The mean animal level prevalence in client herds as reported by veterinarians (practicing in the same 9 states as surveyed producers) was 5% (0, 2, 60). The mean percentage of client herds with at least one infected animal was 26% (0, 10, 100). The mean percentage of infected cows within infected herds was 9% (0.01, 5, 80) (Table 4). The percentage of client herds with at least one infected cow was higher for JD certified veterinarians (P < 0.001) and veterinarians that had performed a risk assessment (P = 0.010). The methods used by veterinarians to derive these estimates were the number of animals with clinical signs suggestive of JD (73%, 237/325), ELISA results (52%, 169/325), fecal culture results (31%, 103/325), and an extrapolation from regional or national data (16%, 51/325).

**Table 4:** **Burden of *Mycobacterium avium *** **subspecies *paratuberculosis *** **infection in cow-calf operations reported by the US cow-calf veterinarians**

**Respondent type**	**Responses**		**Median (IQR) of non zero responses (%)**	**Range of all responses (%)**	**P**^**a**^
	**Total**	**Non-zeros**			
**Cattle infected with MAP in practice clientele**					
JD certified	109	105	2 (1–5)	0–60	0.483
Not certified	162	154	2 (1–5)	0–30	
Performed risk assessment	111	109	2 (1–5)	0–50	0.723
Did not perform risk assessment	154	144	3 (1–5)	0–60	
**Client herds with animal(s) infected with MAP**					
JD certified	110	106	20 (5–50)	0–100	0.006
Not certified	166	156	10 (3–34)	0–100	
Performed risk assessment	112	110	15 (5–50)	0–100	0.010
Did not perform risk assessment	158	146	10 (2–50)	0–100	
**Infected cattle within MAP infected client herd**					
JD certified	107	107	5 (3–10)	1–80	0.454
Not certified	154	154	5 (2–10)	0.01–40	
Performed risk assessment	111	111	5 (2–10)	0.05–80	0.268
Did not perform risk assessment	143	143	5 (3–10)	0.01–70	

^a^Wilcoxon rank-sum test based on all responses.

### Management and testing

Although 95% of producers had tested their herds at least once, only 30% of producers with clinical animals in their herd tested purchased additions, 35% weaned calves early from test positive dams, and 12% removed calves from JD suspect dams. Compared to veterinarians’ perceptions, producers were less likely to cull cows with signs consistent with JD (P = 0.003), but more likely to test purchased additions (P = 0.007, Table 5). Veterinarians reported that only 8% of their clients tested purchased additions for JD.

**Table 5:** Frequency of activities performed by cow-calf producers with clinical cattle and perceived by veterinarians to be performed by producers with the sole or a partial intent of controlling Johne’s disease

**Activities to control diseases:**	**Respondent**	**Yes**	**No**	**Odds ratio**	**95% CI**	**P**^**a**^
Remove calves from dams suspected of being infected with MAP prior to nursing	Producers	3	23	1.33	0.37 to 4.80	0.642
	Veterinarians	21	214			
Cull cows showing signs consistent with a diagnosis of Johne’s disease prior to testing	Producers	15	7	0.48	0.18 to 1.26	0.154
	Veterinarians	165	37			
Cull cows with signs consistent with a diagnosis of Johne’s disease after laboratory testing	Producers	19	7	0.19	0.07 to 0.52	0.003
	Veterinarians	240	17			
Cull calves from dams suspected or confirmed to be infected with MAP based on testing or clinical signs	Producers	15	9	1.44	0.60 to 3.46	0.419
	Veterinarians	105	91			
Early weaning of calves from dams with positive results from Johne’s disease tests	Producers	7	13	1.82	0.68 to 4.83	0.245
	Veterinarians	45	152			
Cull cows without clinical signs consistent with a diagnosis of Johne’s disease, but positive serology (ELISA)	Producers	14	8	1.96	0.79 to 4.88	0.153
	Veterinarians	92	103			
Test purchased additions for Johne’s disease	Producers	6	14	5.19	1.77 to 15.24	0.007
	Veterinarians	17	206			

^a^Mid-point exact P values.

Miscellaneous free-text comments from two producers indicated that they purchased only pre-tested cattle from trusted or test-negative herds. One producer not only tested every purchased addition but also segregated those animals for 6 months. Producers were not specifically asked about the disposition of cows with clinical signs consistent with JD, but three producers noted that they culled such animals. A producer also reported that all suspect cows were destroyed. Regarding the disposition of calves, one producer marketed calves from infected dams as feeder cattle rather than retaining them as replacements. Another producer raised replacement heifers separately until 3 years of age. One producer noted that breeders should be encouraged, if not required, to only sell bulls and replacement females that are from test-negative dams. Another producer had a strong belief that herd transmission is due to the reuse of palpation gloves during pregnancy diagnosis. One producer also stated that their management and facilities were designed to control JD by elevating feed bunks and reducing stocking density within winter feeding areas.

The veterinarian (n = 277) recommended protocols for the initiation of a testing program in beef cow-calf herds were bacterial culture of feces (3%), PCR (14%), ELISA (35%) and a combination of these tests (47%). The recommended interval between testing was 12 months by 79% (198/252) of respondent veterinarians. Eleven percent (28/252) recommended testing every six months while one respondent recommended testing more frequently than 6 months. Three percent (8/252) of veterinarians recommended 18 months, 7% (18/252) recommended a testing interval of more than 18 months, and 3% (7/252) of respondents did not believe periodic testing was necessary. A total of 46% (124/270) of respondents preferred a combination of ELISAs with PCR or fecal culture for herds starting a testing program. Thirty-four percent (42/136) of respondents preferred a combination of tests based on the assumption that it would improve sensitivity, specificity or “accuracy”. Thirty-six percent (97/270) of veterinarians preferred ELISA because it is rapid (49%, 48/97), inexpensive (46%, 45/97), and convenient (38%, 37/97) for whole herd screening.

## Discussion

Seedstock producers that had a risk assessment for JD performed in their herds were more likely to be currently enrolled in a JD control program. This can be related to an overall awareness of seedstock producers concerning the importance of improved herd health for better economic outcomes. The mean value of animals in a seedstock herd is typically higher than those in commercial cow-calf operations. Seedstock producers were also more likely to purchase additions compared to non-seedstock producers and this increases the risk of introducing infected animals.

The producer estimated mean cow-level prevalence was 0.8%. This was derived from the aggregation of their best estimate and is not necessarily based on previous test results. Similarly, a nationwide study estimated that 0.4% of US beef cattle were seropositive [4,8]. Other studies have reported a seroprevalence of 3% [7], 5% [6], and 9% [5] and this suggests that the true prevalence might be 7% to 28% after adjustment for the sensitivity and specificity of available serum ELISA [11]. Producers with infected herds (22%) and the mean percentage of infected herds estimated by veterinarians (26%) were lower than previous field studies. Forty percent of beef herds in Missouri [6], 44% of herds in Texas [7], and 63% of herds in Alabama [5] have been reported as seropositive for JD. The perception that there is a higher percentage of infected herds by veterinarians with JD certification or risk assessment experience might be due to a better understanding of disease burden. Another possible explanation for this perception is that producers suspecting that their herd is infected might seek the services of a JD certified veterinarian. Some differences in perceptions are expected because published reports were based on seroprevalence, while respondents answered based on test results, regional or national data, and experience. Furthermore, veterinarians estimated the prevalence based on a typical client herd rather than the testing of a specific herd. The majority of producers had tested their herd, or a part of the herd in the past, but their responses were not necessarily an accurate representation of this test-based prevalence.

Commercial cow-calf producers included in this survey were less likely to be currently enrolled in a control program compared to producers without commercial cow-calf herds. Commercial cow-calf producers tended to maintain a herd with replacements obtained from within the herd itself (closed herd), which enables producers to minimize new infections from additions. A lower comparative prevalence in closed herds also suggests reduced probability of introduction of infected stock. In a previous study, only 25% of producers perceived significant financial and non-financial benefits associated with participation in the VBJDCP [17] and those results were from producers with an extremely low probability of having MAP infected cows (classification level 4). Although 84% (46/55) of closed herds were already reported to be infected, maintaining a closed herd is an effective method to prevent further introduction of infected cows. Mixing replacement heifers from different production units without known low-risk status might increase exposure and chance of transmission from subclinically infected animals [18,19]. Excluding dairy cattle and ensuring more effective farm biosecurity will reduce the risk of introducing MAP infection into beef herds [20]. Strict biosecurity by not allowing infected cattle to enter the farm is the only necessary control measure for uninfected herds [21].

Compared to small herds (< 50 head), test-based prevalence and producers’ estimate of the herd prevalence were higher in medium sized herds (50–149 head) and highest in large (≥ 150 head) herds. The odds of having a cow with clinical signs of JD were higher in larger herds, possibly due to an increased probability of detecting rare events in larger herds. Larger herds might also be more likely to have a higher MAP infection prevalence because of an expected higher rate of animal movements and increased density of animals in areas where cattle congregate for feed, water, and shade [18]. Only 63% (100/160) of producers answered the question concerning having a cow with clinical signs suggestive of JD during the previous year. The true percentage of herds with clinical animals might have been lower than estimated because producers who left the answer blank (handled as missing data) might have had no clinical animals. Most herds reported having only a single case during the previous year.

The number of producers employing herd testing was higher among those enrolled in a control program. Ninety-five percent of herds were tested for MAP at least once although only 74% percent of producer respondents were currently enrolled in a control program. Disinclination from regular testing might be attributed to the low sensitivity of available tests [22,23]. One respondent producer commented that until a test is developed that is more than “50% accurate” it would be “stupid and irresponsible” to perform the management practices proposed in the questionnaire. While it is true that test sensitivities are low, perhaps this illustrates that producers do not have an adequate understanding of test interpretation and the general importance of herd hygiene.

Responses from veterinarians regarding testing were mostly in favor of ELISA for herds starting a control program. Fecal culture is considered more sensitive and specific than ELISA [11,24] although it is costly and there is a long delay in the availability of results due to the slow growth of MAP in culture [25]. The majority of respondent veterinarians recommended a 12-month interval similar to the VBJDCP [13,26,27]. Specific client and herd needs might warrant a different test type and interval. One producer implemented 6-monthly intervals when testing was subsidized by the program. In Canadian dairy herds, ELISA-seropositivity was significantly associated with “open heifers purchased during the last 12 months” [28]. However, testing purchased additions was a preferred practice reported only by 30% of producers and 8% of veterinarians. One of the most important routes of transmission between herds is the addition of subclinically infected animals [18]. While producers in general appeared less concerned about infected replacements, one respondent tested and quarantined additions for 6 months. One of the reasons for not testing additions was that some producers only purchased animals from herds with known JD low-risk status. In Canada, veterinarians perceived that less than half of beef producers would prefer purchasing replacements from herds where a risk assessment had been performed [29].

The immediate removal of calves from an infected dam was only practiced by 12% of producers and perceived to be performed by 9% of veterinarians. Cow-to-calf transmission occurs most commonly within the calving area and early removal would be expected to reduce the risk of transmission [23,30]. Producers within the VBJDP are educated concerning possible routes of cow-to-calf transmission during enrollment into the program [27]. More than half of the responses from both producers and veterinarians favored culling calves based on a positive test or suspect clinical signs in the dam. While calves from test positive dams are reported to have lower weaning weights [31], one producer indicated that such calves can still be fattened and sent to slaughter, which would be economically more rewarding than immediate removal.

A major limitation of this study is that questionnaires were sent only to producers that had on-farm risk assessments performed and herd management plans developed. Also, the response proportions of 17% and 31% from producers and veterinarians, respectively indicates a possibility of non-response bias. The impact of these potential biases could not be assessed because information regarding non-responders was not available. Comparability was attempted by recruiting producers and veterinarians from the same 9 states. However, it is possible that respondent veterinarians might not have served the specific producers included in this study. Only producers with clinical cows during the previous year were included in the analysis of disease control activities because such activities would not be applicable to JD-free herds. However, only 27 respondents reported having had clinical animals in their herd and this caused a lower precision in estimated measures of association.

The percentage of producers currently enrolled in a control program among the responders of this study (75%) is quite high because the DJCs in each state contacted the producers that had risk assessments performed in their herds. Therefore, these producers presumably have more knowledge concerning JD than US beef producers in general. The power to detect significant associations was low for some variables and several significant associations might be counterintuitive. Seedstock producers were more likely to have uninfected herds even though they were more likely to introduce new animals onto their premises (maintain an open rather than closed herd). A possible explanation is that infected seedstock producers do not adopt a formal control program because they fear lack of confidentiality.

## Conclusions

Seedstock producers that had JD risk assessments performed on their farms were more concerned with JD compared to other producer groups. These producers were less likely to have an infected herd and more likely to enroll in a control program despite the fact that they purchased additions more frequently than other producer categories. Testing purchased additions was not a popular practice. Serum ELISA based culling was also not common among producers that had clinical animals in the past. This is further supported by the fact that closed herds had a lower perceived prevalence, lower test positive percentage, and also a lower risk of having animals with clinical JD. Educational activities regarding better biosecurity measures and control of JD should be directed at larger herds since those were more likely to have test positive cows.

## Abbreviations

JD: Johne’s disease; DJC: Designated JD Coordinator is the person selected by the state animal health official and the Area Veterinarian in Charge; JD certified veterinarian: Accredited veterinarian that received additional training on JD epidemiology, sample collection, testing, test interpretation, state and federal program requirements, on-farm risk assessments, and herd management plan development.; MAP: *Mycobacterium avium* subsp. *paratuberculosis*; VBJDP: US Voluntary Bovine Johne’s Disease Control Program. This program was developed in cooperation with the National JD Working Group and the JD committee of the United States Animal Health Association, State Veterinarians and industry representatives. It was approved by the USDA’s Animal and Plant Health Inspection Service (APHIS), Veterinary Services (VS). The Program was administered at the individual state level and supported by industry and the federal government.

## Competing interests

The authors declare that they have no competing interests.

## Authors’ contributions

BB drafted the questionnaires, recorded the responses, analyzed the data, and prepared the manuscript draft. GTF, JBO, AJR and SCP participated in the design of both the questionnaires. AJR coordinated the design and deployment of both questionnaires. BB, GTF and JBO conducted the statistical analysis. All authors participated in development of the manuscript. All authors read and approved the final manuscript.
